# Unleashing Ferroptosis in Human Cancers: Targeting Ferroptosis Suppressor Protein 1 for Overcoming Therapy Resistance

**DOI:** 10.3390/antiox12061218

**Published:** 2023-06-05

**Authors:** Jaewang Lee, Jong-Lyel Roh

**Affiliations:** Department of Otorhinolaryngology-Head and Neck Surgery, CHA Bundang Medical Center, CHA University, Seongnam, Gyeonggi-do 13496, Republic of Korea

**Keywords:** ferroptosis, ferroptosis suppressor protein 1, FSP1 inhibitor, cancer, therapy

## Abstract

Ferroptosis, a recently identified form of regulated cell death characterized by the iron-dependent accumulation of lethal lipid peroxidation, has gained increasing attention in cancer therapy. Ferroptosis suppressor protein 1 (FSP1), an NAD(P)H-ubiquinone oxidoreductase that reduces ubiquinone to ubiquinol, has emerged as a critical player in the regulation of ferroptosis. FSP1 operates independently of the canonical system xc^–^/glutathione peroxidase 4 pathway, making it a promising target for inducing ferroptosis in cancer cells and overcoming ferroptosis resistance. This review provides a comprehensive overview of FSP1 and ferroptosis, emphasizing the importance of FSP1 modulation and its potential as a therapeutic target in cancer treatment. We also discuss recent progress in developing FSP1 inhibitors and their implications for cancer therapy. Despite the challenges associated with targeting FSP1, advances in this field may provide a strong foundation for developing innovative and effective treatments for cancer and other diseases.

## 1. Introduction

Ferroptosis is a distinctive form of cell death characterized by the iron-dependent accumulation of lipid peroxides [1]. An oncogenic RAS-selective lethal chemical erastin was found to induce ferroptosis and was instrumental in the initial discovery of this form of cell death in 2012 [2]. While the exact mechanisms of ferroptosis induction are still being studied, it has been suggested that ferroptosis may originate from oxytosis, which is a type of neural cell death triggered by glutamate toxicity that inhibits cystine uptake through the cystine/glutamate antiporter system xc^–^ (xCT), ultimately leading to glutathione (GSH) depletion and oxidative stress [3]. Furthermore, oxytosis has been identified as a novel form of cell death that involves lipoxygenase (LOX) activation [4]. Oxytosis was further characterized as cell death resulting from the loss of glutathione peroxidase 4 (GPX4), which induces lipid peroxidation and requires 12/15-LOX activity and apoptosis-inducing factor mediation [5]. Recently, the apoptosis-inducing factor mitochondria-associated 2 (AIFM2), initially described as a proapoptotic gene, was later renamed ferroptosis suppressor protein 1 (FSP1) and identified as a potent GSH-independent protection system that prevents ferroptotic cell death [6]. Ferroptosis has gained significant attention in the scientific community due to its unique morphological and biochemical features, distinguishing it from other cell death forms. In 2018, ferroptosis was formally recognized as a distinct form of cell death, triggered by intracellular oxidative and iron imbalances [7]. This iron-dependent vulnerability leads to lipid peroxidation, distinguishing ferroptosis from other programmed cell death pathways, such as apoptosis. However, the classification of ferroptosis as a regulated cell death pathway has been a topic of discussion and remains an area of ongoing research.

**Figure 1:**
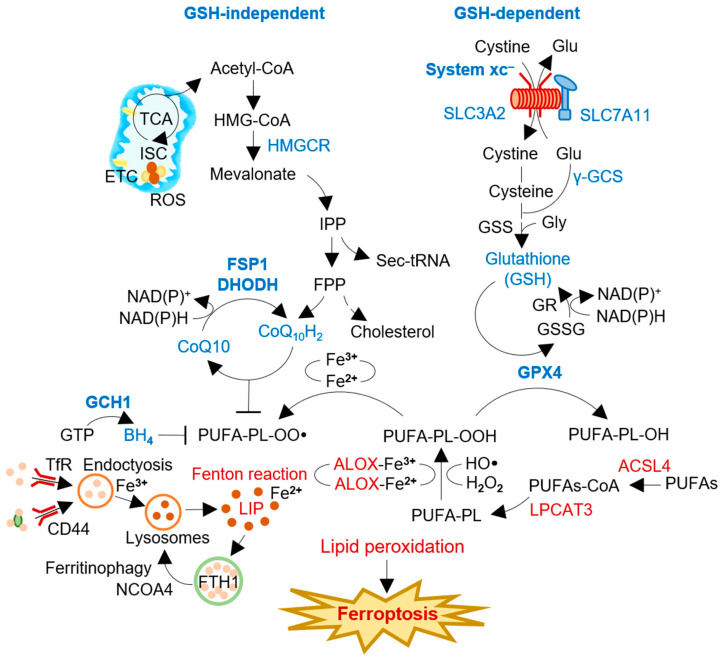
The process and regulators of ferroptosis induction. Intracellular labile iron pool retains chelatable redox-active ferrous iron (Fe^2+^) that can generate soluble radicals via the Fenton reaction. Radical-trapping antioxidant (RTA) systems can inhibit the propagation of lipid peroxidation and protect cells from excessive lipid peroxidation. System xc^–^ and GPX4 are involved in the GSH-dependent canonical pathway, negatively regulating ferroptosis against membrane lipid peroxidation. FSP1, DHODH, and GCH1 are GSH-independent RTAs using lipophilic radical scavengers of ubiquinol or BH_4_. FSP1 and DHODH reduced ubiquinone (CoQ_10_) to ubiquinol (CoQ_10_H_2_) with the consumption of NAD(P)H. The regulators inhibit (blue) or promote (red) ferroptosis induction in cancer cells. ACSL4, acyl-CoA synthetase long chain family member 4; ALOX, arachidonate lipoxygenase; BH4, tetrahydrobiopterin; CoA, coenzyme A; DHODH, dihydroorotate dehydrogenase; FPP, farnesyl pyrophosphate; FSP1, ferroptosis suppressor protein 1; GCH1, guanosine triphosphate cyclohydrolase 1; γ-GCS, γ-glutamylcysteine synthetase; Glu, glutamate; GPX4, glutathione peroxidase 4; GSH, glutathione; GSS, glutathione synthatase; GSSG, glutathione disulfide; HMGCR, 3-hydroxy-3-methylglutaryl-CoA reductase; HO•, hydroxyl radical; IPP, isopentenyl pyrophosphate; ISC, iron-sulfur cluster; LIP, labile iron pool; LPCAT3, lysophosphatidylcholine acyltransferase 3; NADP, nicotinamide adenine dinucleotide phosphate; NCOA4, nuclear receptor coactivator 4; PL-OO•, lipid peroxyl radical; PUFA-PL, polyunsaturated fatty acid-containing phospholipid; PL-PUFA-OH, polyunsaturated fatty acid-containing phospholipid alcohol; PUFAs, polyunsaturated fatty acids; ROS, reactive oxygen species; Sec-tRNA, selenocysteine-tRNA; TCA, tricarboxylic acid cycle; TfR, transferrin receptor; xCT, system xc^–^ cystine/glutamate antiporter.

Ferroptosis is characterized by the accumulation of lipid peroxides and redox-active iron, leading to oxidative damage of cellular membranes and ultimately causing cell death (Figure 1) [1,2]. The intracellular labile iron pool plays a crucial role in ferroptosis. It retains the most chelatable redox-active ferrous iron (Fe^2+^), which generates highly reactive radicals through the Fenton reaction [8]. Iron-dependent enzymes such as 12/15-LOX, P450 oxidoreductase, and prostaglandin-endoperoxide synthase 2 catalyze the reaction between ferrous iron and polyunsaturated fatty acid (PUFA)-PLs, which leads to excessive lipid peroxidation and, ultimately, causes damage to cellular membranes [9]. The esterification and incorporation of arachidonate to PLs in the cellular membrane by acyl-CoA synthetase long-chain family member 4 (ACSL4) and lysophosphatidylcholine acyltransferase 3 (LPCAT3) are required for ferroptosis [10]. Peroxisomes, cytoplasmic organelles with membrane-bound oxidative properties, are critically involved in ferroptosis through the biosynthesis of plasmalogens, also known as ether lipids [11]. The study emphasizes the importance of peroxisome-dependent plasmalogen generation as a vulnerable pool of ether lipids that can undergo oxidative damage and contribute to ferroptosis in various cell types, such as cancer cells, neurons, and cardiomyocytes. However, radical-trapping antioxidant (RTA) systems can inhibit the propagation of lipid peroxidation and protect cells from excessive lipid peroxidation. [12]. GPX4 and system xc^–^ are two key regulators of ferroptosis, which have primary antioxidant functions in detoxifying cellular lipid peroxidation [13].

The involvement of redox-active iron is essential for oxidative damage to membrane lipids in the ferroptosis process [1]. The labile iron pool, a chelatable and redox-active fraction of cellular iron, plays a critical role in cellular iron metabolism [14]. Although a small fraction of the total cellular redox-active iron, cytosolic labile iron plays a crucial role in cellular iron metabolism [15]. Iron homeostasis is regulated through iron uptake via transferrin receptor 1 (TFR1) and its sequestration into ferritins. CD44, a cell surface marker, also facilitates the endocytosis of iron-bound hyaluronates through its interaction with hyaluronates, contributing to iron homeostasis [16]. CD44 contributes to the internalization of both iron and copper. Salinomycin, an antimicrobial agent with demonstrated effects on iron-addicted cancer stem cells, sequesters iron within lysosomes [17,18]. This sequestration leads to a decrease in intracellular iron levels, activating autophagy and resulting in the release of iron through ferritin degradation [19]. The degradation of ferritin during the autophagy process, known as ferritinophagy, is facilitated by nuclear receptor coactivator 4 (NCOA4), selectively enriched in autophagosomes [20]. NCOA4-mediated ferritinophagy plays a critical role in initiating ferroptosis [21]. Furthermore, ferroptosis has been identified as an autophagic cell death process induced by NCOA4-mediated ferritinophagy [22,23].

Preventing ferroptosis by inhibiting lipid peroxyl radicals is not limited to the canonical GPX4 and system xc^–^ antioxidant system. Coenzyme Q_10_ (CoQ_10_) can act as a lipid peroxide scavenger and electron transporter [24]. FSP1 and dihydroorotate dehydrogenase (DHODH) are enzymes that reduce CoQ_10_ (ubiquinone) to CoQ_10_H_2_ (ubiquinol) (Figure 1). FSP1 localizes to the plasma membrane through N-terminal myristoylation and can inhibit ferroptosis by functioning as a radical scavenger of lipid peroxides and recycling vitamin E [6,25]. Similarly, DHODH in the inner mitochondrial membrane also contributes to the reduction of CoQ_10_ [26]. FSP1 and DHODH play a role in preventing ferroptosis, implicating them as promising targets for cancer therapy, particularly in cancers with relatively less functioning of the core ferroptosis regulatory system, xCT/GSH/GPX4 [27]. As a promising target for ferroptosis-based cancer therapy, the depletion of reduced CoQ_10_ has recently gained attention [28]. Thus, understanding the role of FSP1 in ferroptosis and its modulation, as well as exploring the potential of targeting FSP1 for ferroptosis induction in cancer therapy, is crucial. This review aims to provide a comprehensive overview of FSP1 and ferroptosis, emphasizing the importance of FSP1 modulation and its potential as a therapeutic target in cancer treatment.

## 2. Understanding the Structure and Function of FSP1

**Figure 2:**
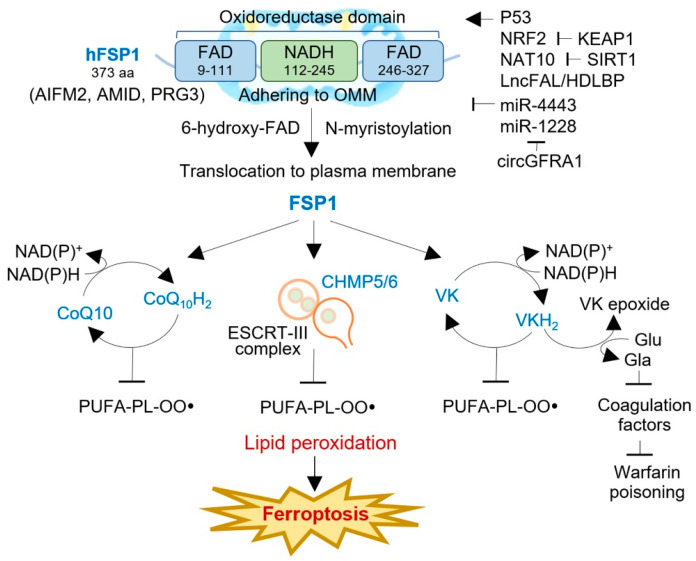
The structure, function, and regulation of FSP1 in human cancers. FSP1 is a NAD(P)H-ubiquinone oxidoreductase, composing FAD- and NADH-dependent domains with an N-terminal myristoylation motif. FSP1 adheres to the outer mitochondrial membrane (OMM) and, after N-myristoylation, is translocated to the plasma membrane or lipid droplets, where it interacts with 6-hydroxyl-FAD. FSP1 exerts ferroptosis resistance via three distinct mechanisms: the FSP1-CoQ_10_-NAD(P)H pathway, the FSP1-ESCRT-III-dependent membrane repair pathway, and the FSP1-VKH_2_-NAD(P)H pathway. The activity of FSP1 is regulated by multiple factors, e.g., p53, NRF2, NAT10, LncFAL, miR-4443, mi-1228, and circGFRA1. AIFM2, apoptosis-inducing factor mitochondria-associated 2; AMID, AIF-homologous mitochondrion-associated inducer of death; CHAMP, charged multivesicular body protein; ESCRT, an endosomal sorting complex required for transport; FAD, flavin adenine dinucleotide; Gla, γ-carboxyglutamate; KEAP1, Kelch-like ECH-associated protein 1; NAT10, N-acetyltransferase 10; lncFAL, ferroptosis-associated long non-coding RNA; NRF2, nuclear factor erythroid 2-related factor 2; PRG3, p53-responsive gene 3; SIRT1, sirtuin 1; VK, vitamin K; VKH_2_, hydroquinone.

Apoptosis-inducing factors (AIFs) are a group of flavoproteins that can trigger caspase-independent apoptotic cell death [29]. In humans, there are three AIF isozymes: AIFM1, AIFM2, and AIFM3. AIFM1 is the most abundant isozyme and is initially translated into the cytosol before being transported to the mitochondrial membrane [30]. There, it undergoes folding and acquires its functional structure with the help of flavin adenine dinucleotide (FAD). In contrast, AIFM2 (FSP1) lacks a mitochondrial targeting sequence, causing no entrance into the mitochondria and adherence to the outer mitochondrial membrane (OMM), and features an N-terminal myristoylation motif [31]. FSP1 consists of a short hydrophobic region at the N-terminus and a FAD-dependent oxidoreductase domain [32]. Studies have shown that the myristoylation motif is critical for targeting FSP1 to lipid droplets and the plasma membrane, where it interacts with 6-hydroxy-FAD to exert its function (Figure 2) [25]. FSP1 is also associated with mitochondria to induce apoptosis. Deletion mutations at the N-terminal region (aas 1–185 and 1–300) result in nuclear localization and failure to affect cell death [33]. Additionally, domain mapping experiments have revealed that only the C-terminal 187 aa of FSP1 is required for apoptotic induction, whereas mutations in the N-terminal domains responsible for its oxidoreductase function do not affect its apoptotic function [34]. Interestingly, the AIFM2 gene contains a putative p53-binding element in intron 5, suggesting that p53 can activate its gene expression [31,35]. Overall, FSP1 plays an essential role in apoptosis and ferroptosis suppression. Further studies are needed to fully understand its complex regulatory mechanisms and potential as a therapeutic target for cancer treatment.

FSP1 is a flavoprotein and NAD(P)H-dependent oxidoreductase critical in suppressing ferroptosis. By reducing ubiquinone-10 to ubiquinol-10 at the plasma membrane, using N-myristoylation and NAD(P)H as a substrate, FSP1 inhibits ferroptosis in a GPX4- and GSH-independent manner [6,25]. This reduction of oxidized CoQ10 decreases the pool of oxidized CoQ10, acting as a lipophilic RTA that stops the propagation of lipid peroxides, thus preventing ferroptosis (Figure 2). To confirm the anti-ferroptotic effect of FSP1, co-autoxidation experiments were conducted with egg phosphatidylcholine and STY-BODIPY, which uses a lipophilic alkoxyl radical generator. Additionally, FSP1 can indirectly generate α-tocopherol via CoQ_10_H_2_ or directly produce it in vitro, providing a reactive RTA effect [6]. Interestingly, the acute reduction in cellular CoQ levels caused by 4-chlorobenzoic acid (4-CBA) or CoQ_2_ knockout does not significantly affect the sensitivity to RSL3 as much as FSP1 knockout does, indicating that other FSP1-mediated pathways may contribute to ferroptosis resistance [25]. These findings suggest that FSP1 is a promising therapeutic target for tumors responsive to FSP1 inhibitors.

Researchers conducted a counter-screen experiment on FSP1-overexpressing cells in both wild-type and GPX4 knockout settings, which led to the discovery of a potent FSP1 inhibitor called iFSP1. The strong protective effect of FSP1 on GPX4 knockout cells was the basis for identifying iFSP1 [6]. Previous studies have shown that the first generation of potent FSP1 inhibitors can induce ferroptosis in various tumor cells, and iFSP1 treatment effectively sensitized cancer cells to ferroptosis. In addition, the expression of AIFM2 positively correlates with resistance to GPX4 inhibitors in cancer cell lines, suggesting that FSP1 inhibitors have potential utility as an alternative therapeutic strategy [25]. It is worth noting that the withdrawal of the ferroptosis inhibitor ferrostatin-1 reduced tumor growth in the FSP1/GPX4 double knockout but not the GPX4 single knockout in the H460 lung cancer mouse xenograft model, indicating that targeting FSP1 may be a promising therapeutic strategy to overcome resistance to ferroptotic cell death in these clinical contexts [25].

FSP1 has recently been identified as a crucial suppressor of ferroptotic cell death by reducing the concentration of CoQ_10_ after treatment with erastin, sorafenib, and RSL3 [36]. Interestingly, the exogenous administration of CoQ_10_ failed to reverse ferroptotic cell death in FSP1-silenced cells, indicating that other mechanisms are involved in ferroptosis resistance. A recent study has shown that FSP1 regulates the expression of the charged multivesicular body protein 5 (CHMP5) and CHMP6, essential subunits of the endosomal sorting complex required for transport (ESCRT)-III-dependent membrane repair machinery [36]. Knocking down FSP1 suppressed the expression of the CHMP5 and CHMP6 induced by the RSL3 treatment, whereas the overexpression of the CHMP5 restored cell viability and rescued cells from RSL3-, erastin-, and sorafenib-induced cell death in both wild-type and FSP1-silenced cells. These findings suggested that ESCRT-III-dependent membrane repair is another mechanism that underlies FSP1-mediated ferroptosis resistance.

FSP1 functions as a vitamin K reductase, preventing lipid peroxidation by reducing vitamin K to its corresponding hydroquinone (VKH_2_) [37]. Vitamin K is a redox-active naphthoquinone that resembles ubiquinone, the oxidized form of CoQ [38]. The canonical vitamin K cycle involves the conversion of vitamin K to VKH_2_, which acts as a potent reactive thiol antioxidant [39]. FSP1 efficiently reduces vitamin K to VKH_2_, a potent reactive thiol antioxidant that prevents lipid peroxidation [40]. Three types of naturally occurring vitamin K compounds (phylloquinone, menaquinone-4 (MK4), and menadione) protected cells from the ferroptosis induced by GPX4 deletion [37]. All three types of vitamin K also rescued ferroptosis caused by ferroptosis inducers and glutamate-induced neuronal ferroptosis but did not protect against apoptosis, necroptosis, or pyroptosis. FSP1-mediated vitamin K reduction was also responsible for the vitamin K antidotal effect against warfarin poisoning [41]. FSP1 serves as the vitamin K reductase accountable for the warfarin-resistant alternative vitamin K reduction pathway. MK4-treated FSP1^−/−^ mice exhibited a much lower conversion rate of MK4 to MK4 epoxide and significantly prolonged prothrombin time than FSP1^+/−^ mice upon exposure to high doses of warfarin. This result highlighted the critical role of FSP1 in the antidotal effect of high-dose vitamin K against warfarin poisoning [37]. Therefore, FSP1 acts as a vitamin K reductase, producing VKH_2_ via NAD(P)H consumption to prevent lipid peroxidation, maintain a warfarin-resistant non-canonical vitamin K cycle, and inhibit ferroptosis. However, it is essential to note that FSP1 activity varies among individuals, and individuals with high FSP1 activity may have reduced effectiveness of warfarin. In contrast, those with low FSP1 activity require a higher dose of vitamin K to mitigate the risk of warfarin poisoning.

Previous studies suggest that FSP1, also known as AIFM2 or AMID, may have a significant role in signaling mitochondrial stress. When oxidative stress occurs, FSP1 binds with 4-hydroxy-2-nonenal (HNE), a lipid peroxidation end product, forming a lipid adduct that lacks oxidoreductase activity [42]. The HNE-FSP1 adduct is then transported from the mitochondria to the nucleus, leading to DNA damage and cell death. Notably, doxorubicin treatment can increase cardiac levels of HNE and AIFM2. The HNE adduction of AIFM2 inhibits the NADH oxidoreductase activity of AIFM2, promoting its translocation from mitochondria. This discovery reveals an unexpected role of these proteins in mitochondrial stress signaling and the adverse effects of cancer therapy. Apart from its role in mitochondrial stress signaling, FSP1 can bind to nuclear DNA non-specifically [34]. FSP1 can directly bind to nuclear DNA and alter chromatin condensation. Additionally, FSP1 can induce caspase- and p53-independent apoptosis by disrupting mitochondrial morphology and releasing proapoptotic factors [33]. Under stress conditions, such as hypoxia, which activates p53-mediated apoptosis, FSP1 may stabilize p53 by inhibiting its degradation, accelerating the apoptotic process [35]. Under normal cellular conditions, FSP1 may promote cell survival by generating reactive oxygen species (ROS) to maintain survival signaling [43]. FSP1 is crucial in various cellular processes, including mitochondrial stress signaling, nuclear DNA binding, apoptosis induction, and cell survival. However, its exact mechanisms and functions require further investigation to provide a comprehensive understanding of this protein’s complex actions in various cellular processes.

The discovery of FSP1 as a crucial suppressor of ferroptosis has shed light on the intricate molecular mechanisms underlying this form of cell death. FSP1 exerts its protective effects against ferroptosis. These pathways involve the reduction of CoQ10 and vitamin K, as well as the regulation of ESCRT-III-dependent membrane repair, which collectively contribute to inhibiting lipid peroxidation and preventing ferroptosis. However, several unanswered questions remain regarding the regulation of FSP1 expression and activity. Investigating the molecular structure of FSP1 and elucidating the signaling pathways that control its expression and function would provide valuable insights into the precise mechanisms by which FSP1 suppresses ferroptosis.

Additionally, identifying potential therapeutic targets for developing FSP1 activators or inhibitors holds great promise for developing novel treatment strategies for cancer and other diseases associated with ferroptosis. Further research in these areas has the potential to uncover novel therapeutic approaches that specifically target FSP1 and its related pathways, thus enhancing our understanding of ferroptosis and its implications in various diseases. By exploring the intricate mechanisms of FSP1, researchers can pave the way for developing innovative interventions to modulate ferroptosis and improve patient outcomes.

## 3. Regulation of FSP1 Activity in Cancer

FSP1 (AIFM2) was initially identified as a p53 target gene that is induced during p53-dependent apoptosis in human colon cancer cells, and it was referred to as an AIF-homologous mitochondrion-associated inducer of death (AMID) or p53-responsive gene 3 (PRG3) [33]. Further studies have shown that AIFM2 has a proapoptotic function and translocates from mitochondria to the nucleus in response to toxicological stress, where it binds to DNA to promote caspase-independent nuclear apoptosis through its C-terminus [44]. Despite its initial identification as a potential tumor suppressor gene, its downregulation in tumors suggests that AIFM2 may have downstream effects of p53, such as growth arrest or apoptosis, but may not play a critical, non-redundant role in normal development and p53-mediated tumor suppression (Figure 2) [31,35]. In a mouse erythroleukemia cell line study, the knockdown of AIFM2 did not result in a significant difference in cell death compared to control cells but suggested a potential function in cell differentiation [45]. Furthermore, it is essential to address the limitations and discrepancies in the existing research on FSP1 and its regulation in cancer. While FSP1’s role as a p53 target gene has been established, the precise mechanisms governing its expression, activity, and interaction with other signaling pathways in the context of cancer need further investigation. Additionally, the functional significance of FSP1 downregulation in tumors and its impact on cancer progression warrant thorough exploration.

AIFM2 is a protein whose oxidoreductase activity can be modulated to influence its NADH oxidase activity [34]. This protein is associated explicitly with lipid droplets in brown adipose tissue and is induced by cold exposure and diet, where it functions as an NADH oxidase [46]. AIFM2 plays a role in increasing mitochondrial activity, which leads to the production of cytosolic NAD^+^ to support robust glycolysis and electron transport chain to facilitate thermogenesis. To optimize thermogenesis, the glycolytic and glucose oxidation effects of AIFM2 depend on its NADH oxidase activity, which acts as a mammalian external NADH dehydrogenase. AIFM2 can reduce cytochrome c and other electron acceptors, including molecular oxygen, in a NAD(P)H-dependent manner, indicating its NAD(P)H oxidase activity. Although AIFM2 contains a 6-hydroxy-FAD cofactor instead of FAD, studies have shown that NADPH binding is preferred over NADH [33,34]. These studies suggest that the oxidoreductase activity of AIFM2 can be modulated by exogenous DNA binding, which can regulate ROS production. Recent studies have demonstrated that AIFM2 can act as an endogenous suppressor of GSH-independent ferroptosis via its oxidoreductase activity, which regenerates ubiquinone to ubiquinol and traps lipid peroxyl radicals to prevent peroxidative damage to lipids [6,25]. Mutations in AIFM2, such as G156A, do not affect the levels or localization of FSP1, but they can impair the protein’s ability to reduce ubiquinone and its anti-ferroptotic activity [25], further supporting the role of AIFM2 in anti-ferroptosis. However, the effect of naturally occurring mutations such as M135T and D288N has not been studied yet. It is relevant to ferroptosis sensitivity as these mutations are more frequently found in tumors [47]. In summary, AIFM2 exhibits oxidoreductase activity that can be modulated, impacting its NADH oxidase activity and involvement in thermogenesis. Its ability to regenerate ubiquinone and trap lipid peroxyl radicals highlights its role in suppressing GSH-independent ferroptosis. Further research is needed to elucidate the precise mechanisms underlying AIFM2’s function, particularly in the context of naturally occurring mutations and their implications in ferroptosis and tumor biology.

FSP1 is regulated transcriptionally by the nuclear factor erythroid 2-related factor 2 (NRF2), an unstable protein typically targeted for degradation by the E3 ubiquitin ligase KEAP1 (Figure 2). However, under oxidative stress conditions, the degradation of NRF2 is prevented, allowing it to enter the nucleus and bind to the antioxidant response element, thus activating genes related to antioxidant defense and redox maintenance. Kelch-like ECH-associated protein 1 (KEAP1) acts as the primary negative regulator of NRF2 and functions as a tumor suppressor. KEAP1 contains cysteine residues vulnerable to electrophilic attacks [48]. Inactivation of KEAP1 enhances resistance to ferroptosis by stabilizing NRF2 and its target genes, including those involved in the xCT/cysteine/GSH axis, which is vital for ferroptosis inhibition [49]. The inactivation of KEAP1 can also influence genes associated with iron metabolism, GSH biosynthesis, and antioxidant responses, all of which contribute to ferroptosis evasion [50]. Interestingly, a recent study has revealed that NRF2-mediated transcriptional control also targets FSP1 [51]. Frequent inactivation or mutation of KEAP1 is observed in human cancers, and KEAP1-mutant tumors are frequently resistant to conventional therapies [52]. In lung cancer cells, deficiency or mutations in KEAP1 result in upregulating the CoQ–FSP1 axis via NRF2, leading to resistance to ferroptosis and radiation therapy. NRF2 deletion re-sensitizes KEAP1 knockout cells to ferroptosis induced by RSL3 or ML162. Restoring FSP1 expression in KEAP1/NRF2 double knockout cells reinstates ferroptosis resistance in these cells. Targeting the CoQ–FSP1 axis is a promising approach to sensitize KEAP1-mutant lung cancer cells or tumors to radiation therapy by inducing ferroptosis. This could lead to a new therapeutic strategy for addressing ferroptosis in KEAP1-mutant lung cancers.

The N-acetyltransferase 10 (NAT10) can modulate FSP1 mRNA stability and protein expression by modifying mRNA at ac4C sites, unique to NAT10 [53]. NAT10 is the only known enzyme to mediate N4-acetylcytidine (ac4C) modification of mRNA, which affects the stability and translation efficiency of mRNA [53]. NAT10 plays a vital role in activating ribosomal RNA biogenesis, and its activity is inhibited by deacetylation mediated by sirtuin 1 [54]. Aberrant NAT10 expression is associated with the occurrence and prognosis of various cancers [55,56]; the inhibition of NAT10 suppresses tumor progression [57]. The knockdown of NAT10 promotes ferroptosis and inhibits proliferation and metastasis in colon cancer cells, which can be reversed by ferroptosis inhibition both in vitro and in vivo [53]. Therefore, NAT10 may be a promising prognostic and therapeutic target in colon cancer by regulating FSP1 mRNA stability and ferroptosis. These regulatory mechanisms shed light on the complex regulation of FSP1 in cancer, highlighting the involvement of NRF2, KEAP1, and NAT10 in modulating FSP1 expression and activity in the context of ferroptosis and therapeutic responses. Further research is needed to fully understand the precise interactions and potential therapeutic implications of these regulatory pathways.

Non-coding RNAs (ncRNAs) play a crucial role in regulating the expression and activity of FSP1 in human cancers [58]. Various ncRNAs, including small, long, and circular ncRNAs, have been identified to modulate ferroptosis by targeting key genes and signaling pathways [59]. Among them, microRNAs (miRNAs) are small ncRNAs that regulate gene expression at the post-transcriptional level [60]. In non-small cell lung cancer (NSCLC), miR-4443 inhibits the protein expression of methyltransferase-like 3 (METTL3), which is responsible for *N*^6^-methyladenosine (m6A) methylation, leading to upregulation of FSP1 expression and regulation of ferroptosis [61]. Overexpressing miR-4443 can reduce FSP1-mediated ferroptosis induced by cisplatin treatment and targeting miR-4443 can sensitize cisplatin-resistant NSCLC cells to cisplatin. Long non-coding RNAs (lncRNAs) are RNA molecules longer than 200 nucleotides that do not code for proteins but participate in various cellular functions [62]. Ferroptosis-associated lncRNA (lncFAL) directly binds to FSP1 and competitively abolishes Trim69-dependent FSP1 polyubiquitination degradation, thereby reducing the vulnerability of hepatocellular carcinoma cells to ferroptosis [63]. LncFAL expression is positively related to FSP1 and is stabilized by binding to high-density lipoprotein-binding protein (HDLBP), which prevents cholesterol accumulation in cells [64]. Blocking FSP1 expression is essential for the induction of ferroptosis in hepatocellular carcinoma, and lncFAL expression is positively related to FSP1 [63]. Circular non-coding RNAs (circRNAs) are another type of regulatory ncRNA that forms a closed, continuous loop of single-stranded RNA without a 5′ end cap or 3′ end poly(A) tail [65]. CircRNAs regulate gene splicing and transcription, sponging miRNAs or RNA-binding proteins, and even translating proteins/peptides [66]. The miR-1228/FSP1 axis is involved in the suppression of ferroptosis in HER-2-positive breast cancer, facilitated by the action of circGFRA1 [67]. Inhibition of circGFRA1 alleviates miR-1228’s inhibitory effect on the AIFM2 target gene, thereby attenuating the malignant progression of HER-2-positive breast cancer. These findings highlight the potential of targeting circGFRA1 and the miR-1228/FSP1 axis as a promising therapeutic approach for HER-2-positive breast cancer. These studies underscore the significance of small, long, and circular ncRNAs in modulating ferroptosis in human cancers through regulating FSP1, a potent suppressor of ferroptosis. Understanding the intricate interplay between ncRNAs and FSP1 opens new avenues for developing therapeutic strategies targeting ferroptosis in cancer treatment.

## 4. Implications of Targeting FSP1 for Cancer Therapy

AIFM2 has been proposed as a potential biomarker due to its downregulation in most human tumors but upregulation under induced toxicity conditions [31,42,68]. In human lung cancer cells, toxicological stress induces the upregulation of AIFM2 and enhances apoptosis [44]. However, recent studies raise concerns about the pro-tumor growth role of AIFM2 in promoting aerobic glycolysis [46]. Nevertheless, AIFM2 has been implicated in the synergistic proapoptotic effects of some drugs, making it a potential therapeutic target. FSP1 (AIFM2) upregulation in KRAS-mutant cancer cells has been linked to ferroptosis resistance [51,69]. Downstream of KRAS, the MAPK and NRF2 pathways activate FSP1, which protects KRAS-mutant cells from ferroptosis [70]. FSP1 inhibition and ferroptosis-inducing therapy could be a practical approach for KRAS-mutant cancer treatment. The pharmacological targeting of FSP1 may suppress PL peroxidation and ferroptosis through its CoQ_10_-NAD(P)H pathway [6]. FSP1 N-myristylation and stability are associated with ferroptosis inducer and platinum chemotherapy resistance in ovarian cancer [71]. ACSL1, an anti-ferroptosis protein, promotes cancer cell survival and peritoneal seeding by increasing N-myristoylation of FSP1. Cancer cells with low expression of the core system xc^–^/GPX4 pathway are more likely to depend on the CoQ_10_ or other antioxidant systems, making them vulnerable to inhibiting GPX4-independent ferroptosis defense systems [27]. Imbalance ferroptosis defenses in cancer cells can serve as biomarkers to select patients for effective cancer therapies and the optimal application of ferroptosis-inducing compounds [72]. These findings indicate the potential of FSP1 as a therapeutic target and underscore the importance of combinatory therapy that targets FSP1 and additional ferroptosis inducers.

**Figure 3:**
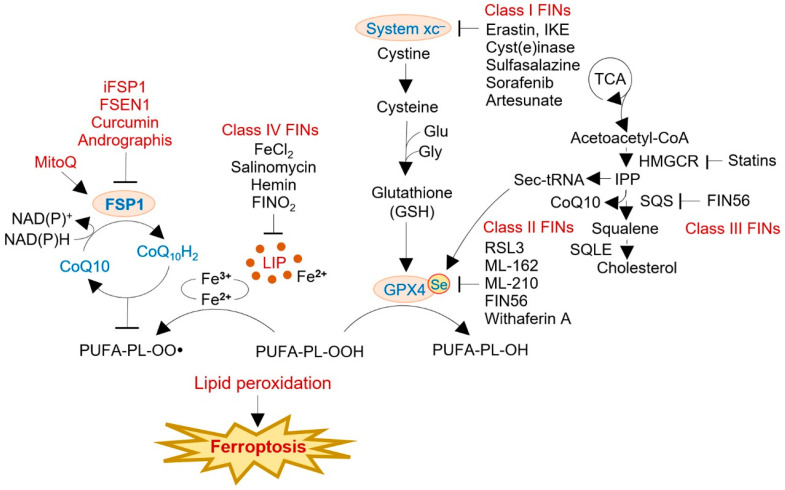
Therapeutic targeting of the FSP1-CoQ_10_ pathway. Inhibition of FSP1 represents a potential strategy for sensitizing cancer cells to current treatments. iFSP1 is a potent FSP1 inhibitor first discovered from a counter-screen of FSP1-overexpressing cells in GPX4 knockout or wild-type backgrounds. The second-generation FSP1 inhibitors have been actively developed. Repurposing existing drugs and natural compounds as ferroptosis inducers also represents an attractive drug discovery. The potential combination of FSP1-targeting agents with ferroptosis-inducing compounds (FINs) or other cancer therapies could be valuable for promoting ferroptosis and treating human cancers. The FINs are classified into I–IV: I, GSH depletion; II, direct GPX4 inhibitors; III, inactivating enzymes in cholesterol synthesis; and IV, inhibiting iron metabolism and its related pathways. MitoQ, mitoquinone mesylate; Se, selenium; SQLE, squalene epoxidase; SQS, squalene synthase.

Pharmacological targeting of FSP1 has emerged as a promising approach in cancer therapy. In a comprehensive study, a library of 10,000 drug-like compounds was screened to identify agents that could effectively induce cell death in cancer cells reliant on FSP1 for survival [6]. This screening process involved monoclonal antibodies, small molecules, and FSP1 knockout to sensitize the cancer cells to ferroptosis inducers. To identify specific inhibitors of FSP1, a counter-screen was performed on FSP1-overexpressing cells in both GPX4 knockout and wild-type backgrounds. This counter-screening strategy led to the discovery of iFSP1, a potent inhibitor of FSP1 activity (Figure 3). It is worth noting that lower expression of FSP1 was observed in 559 cancer cell lines, and this decreased expression was associated with increased dependency on GPX4. Moreover, the level of FSP1 expression directly correlated with resistance to ferroptosis inducers in 860 cancer cell lines. Although targeting GPX4 alone may not achieve optimal anti-tumor effects, simultaneous deletion of both GPX4 and FSP1 has shown promise in effectively reducing tumor growth. FSP1 plays a critical role as a GSH-independent suppressor of PL peroxidation, making it an attractive target for ferroptosis-based cancer therapy. In preclinical studies, the FSP1 inhibitor iFSP1 has been demonstrated to promote ferroptosis in hepatocellular carcinoma and enhance the anti-tumor immune response by stimulating the infiltration of dendritic cells, macrophages, and T cells [73]. Additionally, researchers have explored other strategies for targeting FSP1. For example, a compound called NPD4928, identified from a chemical library, has shown the ability to enhance the cytotoxicity of GPX4 inhibitors in various cancer cells by specifically targeting FSP1 [74]. This approach of combining NPD4928 with GPX4 inhibitors holds promise as a potential therapeutic strategy for inducing ferroptosis in cancer cells. These findings highlight the implications of targeting FSP1 in cancer therapy and underscore the importance of developing combination therapies that simultaneously target FSP1 and utilize other ferroptosis inducers.

Considerable progress has been made in developing second-generation FSP1 inhibitors, addressing the limitations of existing inhibitors, and advancing toward preclinical and clinical stages. A recent preclinical study achieved a significant breakthrough by identifying the binding site of a new FSP1 inhibitor that could selectively target iFSP1-like molecules across different species [75]. This discovery not only sheds light on the binding mechanism but also enables the quantification of direct affinity and competition assays with iFSP1 and three known ligands of human FSP1. These preclinical findings provide a foundation for the rational design and optimization of second-generation FSP1 inhibitors. In addition, several structurally diverse FSP1 inhibitors have been discovered through chemical screens, showing promising preclinical results [76]. Among them, FSEN1 emerged as the most potent inhibitor, exhibiting selective action on FSP1 and sensitizing cancer cells to ferroptosis. Preclinical studies using FSEN1 in combination with endoperoxide-containing ferroptosis inducers, such as dihydroartemisinin, have demonstrated synergistic effects, further supporting its potential as a therapeutic agent. These preclinical findings have identified a range of structurally distinct FSP1 inhibitors that effectively overcome ferroptosis resistance in numerous cancer cell lines. The progress made in preclinical studies paves the way for translating FSP1 inhibitors into clinical trials, offering the potential for novel therapeutic strategies targeting FSP1 in cancer treatment. Further exploration of these inhibitors in clinical settings will be essential to evaluate their safety, efficacy, and potential benefits for patients.

Significant progress has been made in developing FSP1 inhibitors, leading to the discovery of a new class of potent inhibitors with promising anti-tumor effects [73]. These inhibitors selectively target and inhibit the activity of FSP1, resulting in the accumulation of lipid peroxides and the induction of ferroptosis in cancer cells. Notably, when combined with immune checkpoint inhibitors, FSP1 inhibitors have shown enhanced anti-tumor immunity and reduced tumor growth in mouse models, highlighting their potential in combination therapies. Furthermore, FSP1 inhibitors have demonstrated the ability to overcome resistance to ferroptosis in cancer cells resistant to GPX4 inhibitors. This suggests that targeting FSP1 could serve as an alternative strategy for circumventing resistance mechanisms. Recent studies have identified a novel class of FSP1 inhibitors with potent anticancer effects [77]. These inhibitors specifically inhibit FSP1 activity and effectively induce ferroptosis in cancer cells. Notably, FSP1 inhibitors sensitize cancer cells to other ferroptosis inducers, such as erastin and RSL3, further expanding their therapeutic potential. Additionally, when FSP1 inhibitors are combined with GPX4 inhibitors, a synergistic effect is observed in inducing ferroptosis in cancer cells. This combination approach holds promise for overcoming resistance and achieving more effective cancer treatment strategies. These findings highlight the potential of FSP1 inhibitors as a new class of cancer therapeutics, providing a basis for further preclinical and clinical investigations.

Plant-derived compounds have emerged as promising candidates for inducing ferroptosis in cancer cells, and their potential therapeutic effects have been extensively investigated [78]. Curcumin, a well-known chemosensitizer, has been widely studied for its anti-tumor properties and protective effects on normal organs [79]. Recent research has shown that curcumin, as well as Andrographis, can inhibit the negative regulators of ferroptosis, namely, GPX4 and FSP1, in colorectal cancer cells, leading to the activation of the ferroptosis cascade and subsequent anti-tumor effects [80]. Furthermore, the exploration of antioxidant agents targeting FSP1 has revealed intriguing possibilities. Mitoquinone mesylate (MitoQ), an antioxidant specifically targeting mitochondria, has shown promising results in mitigating acetaminophen-induced acute liver injury [81]. Notably, the protective effect of MitoQ against liver injury depends on FSP1 rather than GPX4. The knockdown of FSP1 weakens the protective effect, whereas the knockdown of GPX4 does not have the same impact [82]. In summary, targeting FSP1 holds promise for cancer therapy, as it represents a potential strategy to induce ferroptosis and inhibits tumor growth. Plant-derived compounds, such as curcumin and Andrographis, have shown efficacy in activating ferroptosis. Additionally, antioxidant agents such as MitoQ have demonstrated their dependence on FSP1 for their protective effects against liver injury. Further research and development of compounds targeting these mechanisms are urgently needed to enhance cancer therapy and improve patient outcomes. Preclinical and clinical studies will be essential for evaluating the efficacy and safety of FSP1-targeted treatments and their potential application in diverse cancer types.

This section highlights the potential of targeting FSP1 for cancer therapy, but a more comprehensive analysis of FSP1 inhibitors is needed. The development of FSP1 inhibitors has reached an exciting stage, with ongoing efforts to identify second-generation inhibitors and explore their synergistic effects with other ferroptosis inducers. Combination therapies targeting FSP1 and GPX4, as well as immune checkpoint inhibitors, offer promising avenues for enhancing therapeutic outcomes in cancer treatment. The effectiveness of FSP1 inhibitors should be thoroughly examined, taking into account available studies or preclinical data. Furthermore, it is vital to provide information on the clinical trial stage of these inhibitors, including any potential side effects or toxicity concerns reported in the literature. Additionally, the therapeutic implications of targeting FSP1 for other diseases need further elaboration. Supporting evidence and references should be provided to demonstrate how FSP1 inhibition could be a potential therapeutic strategy for diseases beyond cancer. Further research and clinical investigations are needed to fully understand the potential of FSP1 as a therapeutic target and its applicability in various disease contexts.

## 5. Conclusions and Perspectives

Targeting FSP1 holds great promise as a strategy for cancer therapy, and it may also serve as a valuable biomarker for patient stratification and personalized treatment. However, further investigations are needed to fully understand the precise mechanisms of FSP1 regulation and its interactions with other ferroptosis regulators. Optimization of FSP1-targeting agents, including their specificity, efficacy, and safety profiles, is crucial for their successful translation into clinical applications. Large-scale clinical trials are necessary to validate the clinical utility of FSP1 as a prognostic and predictive biomarker. Additionally, exploring the potential combination of FSP1-targeting agents with other cancer therapies could enhance their therapeutic effects.

Ferroptosis is a complex process regulated by multiple cellular pathways, including the CoQ–FSP1 axis. Recent studies have identified promising FSP1 inhibitors and ferroptosis inducers that show potential as anticancer agents. Repurposing existing drugs and natural compounds as ferroptosis inducers offers an attractive avenue for drug discovery and development. However, challenges such as improving the selectivity and pharmacokinetic properties of FSP1 inhibitors and understanding ferroptosis resistance mechanisms need to be addressed. Moreover, developing effective combination therapies that synergize with FSP1 inhibitors and ferroptosis inducers is essential. The advancements in ferroptosis research provide a solid foundation for developing innovative treatments for cancer and other diseases. Continued research and development efforts will uncover new insights into ferroptosis mechanisms and pave the way for more effective therapeutic strategies.

## Data Availability

The data can be shared up on request.

## Abbreviations

ACSL4, acyl-CoA synthetase long-chain family member 4; AIF, apoptosis-inducing factors; AIFM2, apoptosis-inducing factor mitochondria-associated 2; AMID, AIF-homologous mitochondrion-associated inducer of death; CHAMP, charged multivesicular body protein; circRNAs, circular non-coding RNAs; CoQ, coenzyme Q; CoQ_10_, coenzyme Q_10_ (ubiquinone-10); DHODH, dihydroorotate dehydrogenase; ESCRT, endosomal sorting complex required for transport; FAD, flavin adenine dinucleotide; FSP1, ferroptosis suppressor protein 1; GPX4, glutathione peroxidase 4; GSH, glutathione; HNE, 4-hydroxy-2-nonenal; KEAP1, Kelch-like ECH-associated protein 1; lncRNAs, long non-coding RNAs; lncFAL, ferroptosis-associated lncRNA; LIP, labile iron pool; 12/15-LOX, 12/15-lipoxygenase; LPCAT3, lysophosphatidylcholine acyltransferase 3; MitoQ, mitoquinone mesylate; NADP, nicotinamide adenine dinucleotide phosphate; NAT10, N-acetyltransferase 10; ncRNA, non-coding RNAs; NRF2, nuclear factor erythroid 2-related factor 2; PLs, phospholipids; PRG3, p53-responsive gene 3; PUFA, polyunsaturated fatty acid; ROS, reactive oxygen species; RTA, radical-trapping antioxidant; VK, vitamin K; VKH_2_, hydroquinone; xCT, cystine/glutamate antiporter system xc^–^.

## References

[1] Stockwell B.R., Friedmann Angeli J.P., Bayir H., Bush A.I., Conrad M., Dixon S.J., Fulda S., Gascón S., Hatzios S.K., Kagan V.E. (2017). Ferroptosis: A regulated cell death nexus linking metabolism, redox biology, and disease. Cell.

[2] Dixon S.J., Lemberg K.M., Lamprecht M.R., Skouta R., Zaitsev E.M., Gleason C.E., Patel D.N., Bauer A.J., Cantley A.M., Yang W.S. (2012). Ferroptosis: An iron-dependent form of nonapoptotic cell death. Cell.

[3] Murphy T.H., Miyamoto M., Sastre A., Schnaar R.L., Coyle J.T. (1989). Glutamate toxicity in a neuronal cell line involves inhibition of cystine transport leading to oxidative stress. Neuron.

[4] Tan S., Schubert D., Maher P. (2001). Oxytosis: A novel form of programmed cell death. Curr. Top. Med. Chem..

[5] Seiler A., Schneider M., Förster H., Roth S., Wirth E.K., Culmsee C., Plesnila N., Kremmer E., Rådmark O., Wurst W. (2008). Glutathione peroxidase 4 senses and translates oxidative stress into 12/15-lipoxygenase dependent- and AIF-mediated cell death. Cell Metab..

[6] Doll S., Freitas F.P., Shah R., Aldrovandi M., da Silva M.C., Ingold I., Goya Grocin A., Xavier da Silva T.N., Panzilius E., Scheel C.H. (2019). FSP1 is a glutathione-independent ferroptosis suppressor. Nature.

[7] Galluzzi L., Vitale I., Aaronson S.A., Abrams J.M., Adam D., Agostinis P., Alnemri E.S., Altucci L., Amelio I., Andrews D.W. (2018). Molecular mechanisms of cell death: Recommendations of the Nomenclature Committee on Cell Death 2018. Cell Death Differ..

[8] Galaris D., Barbouti A., Pantopoulos K. (2019). Iron homeostasis and oxidative stress: An intimate relationship. Biochim. Biophys. Acta Mol. Cell Res..

[9] Liang D., Minikes A.M., Jiang X. (2022). Ferroptosis at the intersection of lipid metabolism and cellular signaling. Mol. Cell.

[10] Doll S., Proneth B., Tyurina Y.Y., Panzilius E., Kobayashi S., Ingold I., Irmler M., Beckers J., Aichler M., Walch A. (2017). ACSL4 dictates ferroptosis sensitivity by shaping cellular lipid composition. Nat. Chem. Biol..

[11] Zou Y., Henry W.S., Ricq E.L., Graham E.T., Phadnis V.V., Maretich P., Paradkar S., Boehnke N., Deik A.A., Reinhardt F. (2020). Plasticity of ether lipids promotes ferroptosis susceptibility and evasion. Nature.

[12] Bayır H., Anthonymuthu T.S., Tyurina Y.Y., Patel S.J., Amoscato A.A., Lamade A.M., Yang Q., Vladimirov G.K., Philpott C.C., Kagan V.E. (2020). Achieving Life through Death: Redox Biology of Lipid Peroxidation in Ferroptosis. Cell Chem. Biol..

[13] Hassannia B., Vandenabeele P., Vanden Berghe T. (2019). Targeting ferroptosis to iron out cancer. Cancer Cell.

[14] Kakhlon O., Cabantchik Z.I. (2002). The labile iron pool: Characterization, measurement, and participation in cellular processes. Free Radic. Biol. Med..

[15] Arosio P., Levi S. (2010). Cytosolic and mitochondrial ferritins in the regulation of cellular iron homeostasis and oxidative damage. Biochim. Biophys. Acta.

[16] Müller S., Sindikubwabo F., Cañeque T., Lafon A., Versini A., Lombard B., Loew D., Wu T.D., Ginestier C., Charafe-Jauffret E. (2020). CD44 regulates epigenetic plasticity by mediating iron endocytosis. Nat. Chem..

[17] Mai T.T., Hamaï A., Hienzsch A., Cañeque T., Müller S., Wicinski J., Cabaud O., Leroy C., David A., Acevedo V. (2017). Salinomycin kills cancer stem cells by sequestering iron in lysosomes. Nat. Chem..

[18] Versini A., Colombeau L., Hienzsch A., Gaillet C., Retailleau P., Debieu S., Müller S., Cañeque T., Rodriguez R. (2020). Salinomycin derivatives kill breast cancer stem cells by lysosomal iron targeting. Chemistry.

[19] Asano T., Komatsu M., Yamaguchi-Iwai Y., Ishikawa F., Mizushima N., Iwai K. (2011). Distinct mechanisms of ferritin delivery to lysosomes in iron-depleted and iron-replete cells. Mol. Cell. Biol..

[20] Mancias J.D., Wang X., Gygi S.P., Harper J.W., Kimmelman A.C. (2014). Quantitative proteomics identifies NCOA4 as the cargo receptor mediating ferritinophagy. Nature.

[21] Santana-Codina N., Gikandi A., Mancias J.D. (2021). The role of NCOA4-mediated ferritinophagy in ferroptosis. Adv. Exp. Med. Biol..

[22] Gao M., Monian P., Pan Q., Zhang W., Xiang J., Jiang X. (2016). Ferroptosis is an autophagic cell death process. Cell Res..

[23] Hou W., Xie Y., Song X., Sun X., Lotze M.T., Zeh H.J., Kang R., Tang D. (2016). Autophagy promotes ferroptosis by degradation of ferritin. Autophagy.

[24] Banerjee R., Purhonen J., Kallijärvi J. (2022). The mitochondrial coenzyme Q junction and complex III: Biochemistry and pathophysiology. FEBS J..

[25] Bersuker K., Hendricks J.M., Li Z., Magtanong L., Ford B., Tang P.H., Roberts M.A., Tong B., Maimone T.J., Zoncu R. (2019). The CoQ oxidoreductase FSP1 acts parallel to GPX4 to inhibit ferroptosis. Nature.

[26] Boukalova S., Hubackova S., Milosevic M., Ezrova Z., Neuzil J., Rohlena J. (2020). Dihydroorotate dehydrogenase in oxidative phosphorylation and cancer. Biochim. Biophys. Acta Mol. Basis Dis..

[27] Lei G., Zhuang L., Gan B. (2022). Targeting ferroptosis as a vulnerability in cancer. Nat. Rev. Cancer.

[28] Yu H., Yan J., Li Z., Yang L., Ju F., Sun Y. (2023). Recent trends in emerging strategies for ferroptosis-based cancer therapy. Nanoscale Adv..

[29] Ye H., Cande C., Stephanou N.C., Jiang S., Gurbuxani S., Larochette N., Daugas E., Garrido C., Kroemer G., Wu H. (2002). DNA binding is required for the apoptogenic action of apoptosis inducing factor. Nat. Struct. Biol..

[30] Novo N., Ferreira P., Medina M. (2021). The apoptosis-inducing factor family: Moonlighting proteins in the crosstalk between mitochondria and nuclei. IUBMB Life.

[31] Wu M., Xu L.G., Su T., Tian Y., Zhai Z., Shu H.B. (2004). AMID is a p53-inducible gene downregulated in tumors. Oncogene.

[32] Vabulas R.M. (2021). Ferroptosis-Related Flavoproteins: Their Function and Stability. Int. J. Mol. Sci..

[33] Wu M., Xu L.G., Li X., Zhai Z., Shu H.B. (2002). AMID, an apoptosis-inducing factor-homologous mitochondrion-associated protein, induces caspase-independent apoptosis. J. Biol. Chem..

[34] Marshall K.R., Gong M., Wodke L., Lamb J.H., Jones D.J., Farmer P.B., Scrutton N.S., Munro A.W. (2005). The human apoptosis-inducing protein AMID is an oxidoreductase with a modified flavin cofactor and DNA binding activity. J. Biol. Chem..

[35] Ohiro Y., Garkavtsev I., Kobayashi S., Sreekumar K.R., Nantz R., Higashikubo B.T., Duffy S.L., Higashikubo R., Usheva A., Gius D. (2002). A novel p53-inducible apoptogenic gene, PRG3, encodes a homologue of the apoptosis-inducing factor (AIF). FEBS Lett..

[36] Dai E., Zhang W., Cong D., Kang R., Wang J., Tang D. (2020). AIFM2 blocks ferroptosis independent of ubiquinol metabolism. Biochem. Biophys. Res. Commun..

[37] Mishima E., Ito J., Wu Z., Nakamura T., Wahida A., Doll S., Tonnus W., Nepachalovich P., Eggenhofer E., Aldrovandi M. (2022). A non-canonical vitamin K cycle is a potent ferroptosis suppressor. Nature.

[38] Ivanova D., Zhelev Z., Getsov P., Nikolova B., Aoki I., Higashi T., Bakalova R. (2018). Vitamin K: Redox-modulation, prevention of mitochondrial dysfunction and anticancer effect. Redox Biol..

[39] Shearer M.J., Okano T. (2018). Key pathways and regulators of vitamin K function and intermediary metabolism. Annu. Rev. Nutr..

[40] Vervoort L.M., Ronden J.E., Thijssen H.H. (1997). The potent antioxidant activity of the vitamin K cycle in microsomal lipid peroxidation. Biochem. Pharmacol..

[41] Jin D.Y., Chen X., Liu Y., Williams C.M., Pedersen L.C., Stafford D.W., Tie J.K. (2023). A genome-wide CRISPR-Cas9 knockout screen identifies FSP1 as the warfarin-resistant vitamin K reductase. Nat. Commun..

[42] Miriyala S., Thippakorn C., Chaiswing L., Xu Y., Noel T., Tovmasyan A., Batinic-Haberle I., Vander Kooi C.W., Chi W., Latif A.A. (2016). Novel role of 4-hydroxy-2-nonenal in AIFm2-mediated mitochondrial stress signaling. Free Radic. Biol. Med..

[43] Gong M., Hay S., Marshall K.R., Munro A.W., Scrutton N.S. (2007). DNA binding suppresses human AIF-M2 activity and provides a connection between redox chemistry, reactive oxygen species, and apoptosis. J. Biol. Chem..

[44] Lu J., Chen J., Xu N., Wu J., Kang Y., Shen T., Kong H., Ma C., Cheng M., Shao Z. (2016). Activation of AIFM2 enhances apoptosis of human lung cancer cells undergoing toxicological stress. Toxicol. Lett..

[45] Kojima N., Tanaka Y., Kulkeaw K., Nakanishi Y., Shirasawa S., Sugiyama D. (2015). Apoptosis-inducing factor, mitochondrion-associated 2, regulates Klf1 in a mouse erythroleukemia cell line. Anticancer Res..

[46] Nguyen H.P., Yi D., Lin F., Viscarra J.A., Tabuchi C., Ngo K., Shin G., Lee A.Y., Wang Y., Sul H.S. (2020). Aifm2, a NADH oxidase, supports robust glycolysis and is required for cold- and diet-induced thermogenesis. Mol. Cell.

[47] Tate J.G., Bamford S., Jubb H.C., Sondka Z., Beare D.M., Bindal N., Boutselakis H., Cole C.G., Creatore C., Dawson E. (2019). COSMIC: The catalogue of somatic mutations in cancer. Nucleic Acids Res..

[48] Dinkova-Kostova A.T., Kostov R.V., Canning P. (2017). Keap1, the cysteine-based mammalian intracellular sensor for electrophiles and oxidants. Arch. Biochem. Biophys..

[49] Cao J.Y., Poddar A., Magtanong L., Lumb J.H., Mileur T.R., Reid M.A., Dovey C.M., Wang J., Locasale J.W., Stone E. (2019). A genome-wide haploid genetic screen identifies regulators of glutathione abundance and ferroptosis sensitivity. Cell Rep..

[50] Anandhan A., Dodson M., Schmidlin C.J., Liu P., Zhang D.D. (2020). Breakdown of an ironclad defense system: The critical role of NRF2 in mediating ferroptosis. Cell Chem. Biol..

[51] Koppula P., Lei G., Zhang Y., Yan Y., Mao C., Kondiparthi L., Shi J., Liu X., Horbath A., Das M. (2022). A targetable CoQ-FSP1 axis drives ferroptosis- and radiation-resistance in KEAP1 inactive lung cancers. Nat. Commun..

[52] Taguchi K., Yamamoto M. (2020). The KEAP1-NRF2 system as a molecular target of cancer treatment. Cancers.

[53] Zheng X., Wang Q., Zhou Y., Zhang D., Geng Y., Hu W., Wu C., Shi Y., Jiang J. (2022). N-acetyltransferase 10 promotes colon cancer progression by inhibiting ferroptosis through N4-acetylation and stabilization of ferroptosis suppressor protein 1 (FSP1) mRNA. Cancer Commun..

[54] Liu X., Cai S., Zhang C., Liu Z., Luo J., Xing B., Du X. (2018). Deacetylation of NAT10 by Sirt1 promotes the transition from rRNA biogenesis to autophagy upon energy stress. Nucleic Acids Res..

[55] Liu H.Y., Liu Y.Y., Yang F., Zhang L., Zhang F.L., Hu X., Shao Z.M., Li D.Q. (2020). Acetylation of MORC2 by NAT10 regulates cell-cycle checkpoint control and resistance to DNA-damaging chemotherapy and radiotherapy in breast cancer. Nucleic Acids Res..

[56] Zhang X., Chen J., Jiang S., He S., Bai Y., Zhu L., Ma R., Liang X. (2019). N-acetyltransferase 10 enhances doxorubicin resistance in human hepatocellular carcinoma cell lines by promoting the epithelial-to-mesenchymal transition. Oxidative Med. Cell. Longev..

[57] Ma N., Liu H., Wu Y., Yao M., Zhang B. (2022). Inhibition of N-acetyltransferase 10 suppresses the progression of prostate cancer through regulation of DNA replication. Int. J. Mol. Sci..

[58] Zhang P., Wu W., Chen Q., Chen M. (2019). Non-coding RNAs and their integrated networks. J. Integr. Bioinf..

[59] Wu J., Zhu S., Wang P., Wang J., Huang J., Wang T., Guo L., Liang D., Meng Q., Pan H. (2022). Regulators of epigenetic change in ferroptosis-associated cancer (Review). Oncol. Rep..

[60] Bartel D.P. (2018). Metazoan MicroRNAs. Cell.

[61] Song Z., Jia G., Ma P., Cang S. (2021). Exosomal miR-4443 promotes cisplatin resistance in non-small cell lung carcinoma by regulating FSP1 m6A modification-mediated ferroptosis. Life Sci..

[62] Carlevaro-Fita J., Johnson R. (2019). Global Positioning System: Understanding long non-coding RNAs through subcellular localization. Mol. Cell.

[63] Yuan J., Lv T., Yang J., Wu Z., Yan L., Yang J., Shi Y. (2022). HDLBP-stabilized lncFAL inhibits ferroptosis vulnerability by diminishing Trim69-dependent FSP1 degradation in hepatocellular carcinoma. Redox Biol..

[64] Cheng M.H., Jansen R.P. (2017). A jack of all trades: The RNA-binding protein vigilin. Wiley Interdiscip. Rev. RNA.

[65] He A.T., Liu J., Li F., Yang B.B. (2021). Targeting circular RNAs as a therapeutic approach: Current strategies and challenges. Signal Transduct. Target. Ther..

[66] Zhou W.Y., Cai Z.R., Liu J., Wang D.S., Ju H.Q., Xu R.H. (2020). Circular RNA: Metabolism, functions and interactions with proteins. Mol. Cancer.

[67] Bazhabayi M., Qiu X., Li X., Yang A., Wen W., Zhang X., Xiao X., He R., Liu P. (2021). CircGFRA1 facilitates the malignant progression of HER-2-positive breast cancer via acting as a sponge of miR-1228 and enhancing AIFM2 expression. J. Cell. Mol. Med..

[68] Hsiao C.P., Wang D., Kaushal A., Saligan L. (2013). Mitochondria-related gene expression changes are associated with fatigue in patients with nonmetastatic prostate cancer receiving external beam radiation therapy. Cancer Nurs..

[69] Wu S., Zhu C., Tang D., Dou Q.P., Shen J., Chen X. (2021). The role of ferroptosis in lung cancer. Biomark. Res..

[70] Müller F., Lim J.K.M., Bebber C.M., Seidel E., Tishina S., Dahlhaus A., Stroh J., Beck J., Yapici F.I., Nakayama K. (2023). Elevated FSP1 protects KRAS-mutated cells from ferroptosis during tumor initiation. Cell Death Differ..

[71] Zhang Q., Li N., Deng L., Jiang X., Zhang Y., Lee L.T.O., Zhang H. (2023). ACSL1-induced ferroptosis and platinum resistance in ovarian cancer by increasing FSP1 N-myristylation and stability. Cell Death Discov..

[72] Zhang C., Liu X., Jin S., Chen Y., Guo R. (2022). Ferroptosis in cancer therapy: A novel approach to reversing drug resistance. Mol. Cancer.

[73] Cheu J.W., Lee D., Li Q., Goh C.C., Bao M.H., Yuen V.W., Zhang M.S., Yang C., Chan C.Y., Tse A.P. (2023). Ferroptosis suppressor protein 1 inhibition promotes tumor ferroptosis and anti-tumor immune responses in liver cancer. Cell. Mol. Gastroenterol. Hepatol..

[74] Yoshioka H., Kawamura T., Muroi M., Kondoh Y., Honda K., Kawatani M., Aono H., Waldmann H., Watanabe N., Osada H. (2022). Identification of a small molecule that enhances ferroptosis via inhibition of ferroptosis suppressor protein 1 (FSP1). ACS Chem. Biol..

[75] Xavier da Silva T.N., Schulte C., Alves A.N., Maric H.M., Friedmann Angeli J.P. (2023). Molecular characterization of AIFM2/FSP1 inhibition by iFSP1-like molecules. Cell Death Dis..

[76] Hendricks J.M., Doubravsky C., Wehri E., Li Z., Roberts M.A., Deol K., Lange M., Lasheras-Otero I., Dixon S.J., Bersuker K. (2022). Identification of structurally diverse FSP1 inhibitors that sensitize cancer cells to ferroptosis. bioRxiv.

[77] Tong X., Tang R., Xiao M., Xu J., Wang W., Zhang B., Liu J., Yu X., Shi S. (2022). Targeting cell death pathways for cancer therapy: Recent developments in necroptosis, pyroptosis, ferroptosis, and cuproptosis research. J. Hematol. Oncol..

[78] Wu Z., Zhong M., Liu Y., Xiong Y., Gao Z., Ma J., Zhuang G., Hong X. (2022). Application of natural products for inducing ferroptosis in tumor cells. Biotechnol. Appl. Biochem..

[79] Goel A., Aggarwal B.B. (2010). Curcumin, the golden spice from Indian saffron, is a chemosensitizer and radiosensitizer for tumors and chemoprotector and radioprotector for normal organs. Nutr. Cancer.

[80] Miyazaki K., Xu C., Shimada M., Goel A. (2023). Curcumin and Andrographis exhibit anti-tumor effects in colorectal cancer via activation of ferroptosis and dual suppression of glutathione peroxidase-4 and ferroptosis suppressor protein-1. Pharmaceuticals.

[81] Jiang Q., Yin J., Chen J., Ma X., Wu M., Liu G., Yao K., Tan B., Yin Y. (2020). Mitochondria-targeted antioxidants: A step towards disease treatment. Oxidative Med. Cell. Longev..

[82] He X., Liang S.M., Wang H.Q., Tao L., Sun F.F., Wang Y., Zhang C., Huang Y.C., Xu D.X., Chen X. (2023). Mitoquinone protects against acetaminophen-induced liver injury in an FSP1-dependent and GPX4-independent manner. Toxicol. Appl. Pharmacol..

